# Long-term apple orchard cultivation drives selective accumulation and moderate ecological risk of heavy metals in loess Plateau, China

**DOI:** 10.1038/s41598-026-36342-3

**Published:** 2026-01-19

**Authors:** Haifeng Pan, Zhikun Chen, Guanghua Jing, Weixi Wang, Muhammad Imran, Wenna Bao

**Affiliations:** 1 School of Life and Health Sciences , Huzhou College, Huzhou, 313000 China; 2 Key Laboratory of Soil Resource and Biotech Application, Xi’an Botanical Garden of Shaanxi Province (Institute of Botany Shaanxi Province), Xi’an, 710061 China; 3 School of Biological and Chemical Engineering, Zhejiang University of Science and Technology, Hangzhou, 310023 China

**Keywords:** Long-term cultivation, Heavy metals pollution load, Ecological risk, Fertilization, Atmospheric deposition, Ecology, Ecology, Environmental sciences

## Abstract

The long-term intensive cultivation in apple orchards has led to certain heavy metals accumulation yet the relative contributions of fertilizer inputs versus atmospheric deposition remain poorly resolved. Therefore, it is crucial to assess the effect of long-term cultivation on heavy metal pollution in soils of apple orchards, for safe and sustainable fruit production. Unlike the most studies that analyzed only surface soils, this study compares two soil depths with multiple cultivation-age classes and deep-profile background values, supported by positive matrix factorization (PMF) modeling, to distinguish fertilizer-derived and atmospheric sources. A total number of 128 soil samples were collected from two depths (0–20 and 20–40 cm) in apple orchards of varying ages up to 30 years. Overall, the soil pH was alkaline with high soil organic matter, total nitrogen, total phosphorus, total potassium and heavy metal contents. The concentrations of Hg, Cu, and Pb in orchard soils significantly increased with cultivation age at rates of 0.00165 mg kg^−1^, 0.244 mg kg^−1^, and 0.208 mg kg^−1^ per year (equivalent to 4.34%, 0.88%, and 1.11% per year relative to background levels), respectively, while Zn, As, and Cr showed no significant accumulation over time. The cumulative pollution load index of heavy metals was at moderate level in both depths (1.10 and 1.05 respectively). The single heavy metals pollution load index was variable Pb pollution load was high at 0–20 cm depth and Hg at 20–40 cm depth. The cumulative ecological risk index was at considerable level at both depths. However, the single ecological risk index of Hg was only at moderate level. Hg ecological risk is dominated by atmospheric deposition, while Cu, Pb, Zn, As, and Cr are fertilizer-driven. Ecological risk is based on total concentrations; bioavailability was not measured. The PMF model identified inorganic and organic fertilizers as the major contributing factors in Cu, Zn, As, Pb and Cr accumulation, whereas Hg accumulation was mainly due to atmospheric deposition. The study suggests regulating the use of fertilizers inputs and implementing control and remediation practices for sustainable fruit production in apple orchards.

**Supplementary Information:**

The online version contains supplementary material available at 10.1038/s41598-026-36342-3.

## Introduction

Heavy metals pollution in soil is a complex problem, driven by multiple factors including agriculture intensification, urbanization, and industrialization^1^. Due to the increasing food demand, the agricultural intensification (fertilization, pesticides, fungicides, waste water irrigation and application of sewage sludge) are leading to heavy metals accumulation, which are posing a threat to the soil ecosystem health and food safety^2^. In addition to intensive agriculture inputs, coal combustion and industries also contributes towards heavy metals accumulation in soils through atmospheric deposition^3^. Heavy metal pollution in soils can also be inherent from soil parent material^4–6^.

The organic and inorganic fertilizers are the critical components of intensive agriculture, which contains significant concentration of heavy metals. The key fertilizers in China also contains significant concentration of heavy metals as evidenced by many studies. The concentrations of heavy metals in inorganic-organic compound fertilizers were as: arsenic (As) 1.55–37 mg kg^−1^, mercury (Hg) 0.04–2.30 mg kg^−1^, lead (Pb) 1.43–78 mg kg^−1^, cadmium (Cd) 0.15–7.50 mg kg^−1^, chromium (Cr) 11–213 mg kg^−1^, copper (Cu) 7.74–555 mg kg^−1^, zinc (Zn) 21–2700 mg kg^−1^, and nickel (Ni) 6–244 mg kg^−1^^7–9^. In addition to the above sources Bordeaux mixture and Mancozeb fungicides causes Cu and Zn pollution^10^. The concentrations of heavy metals varied among fertilizer types: As was highest in mono and diammonium phosphates (MAP and DAP), while Hg and Pb were most prevalent in superphosphate (SSP). The concentration of Cd and Cr was highest in water-soluble fertilizers, and organic fertilizers respectively^11^. The concentration of heavy metals varies greatly even among the same type of fertilizers^2,12^.

While the accumulation of heavy metals from fertilizers and pesticides in agricultural soils is well-documented, still there is a significant gap in understanding the long-term effects of continuous cultivation on the soil’s heavy metal load, particularly in surface and subsurface soils of apple orchards^13^. Existing studies have either focused on surface soils or neglected the impact of long-term cultivation for heavy metals buildup^14,15^. Given the lack of studies on long-term cultivation impacts on heavy metal accumulation in apple orchards, particularly in surface and subsurface soils, it becomes crucial to investigate these effects in major apple-growing regions.

Apple production significantly contributes to global economy with millions of tons annually. China is the world’s largest producer and consumer of apples with 48 million metric tons production in 2024–2025 (July–June), more than 3% from the year 2023–2024^16^. In terms of growing area, apple cultivation ranks first in China making it a key fruit industry that has developed rapidly over the years. The major apple growing areas in China are Shandong, Liaoning, Hebei, and Henan Shaanxi, Shanxi, and Gansu provinces^17^. Luochuan county, a major apple producing area in Shaanxi province with over 35,000 hectares of apple orchards^18^, lacks details on its soil heavy metals status. Investigating the heavy metals contents in apple orchard soils with time (cultivation years) and their pollution load and ecological risk could provide valuable information for mitigating heavy metal pollution and sustainable fruit production.

Numerous studies have investigated the heavy metals accumulation in agricultural soils worldwide, research specifically focused on orchard systems reveals several methodological limitations that restrict the reliability of long-term pollution assessments. These limitations are even more evident in previous studies from Chinese apple orchards. First, nearly all earlier Chinese investigations relied solely on surface soils (0–20 cm), thereby missing information on deeper soil layers that can store legacy metal inputs and better reflect natural geochemical backgrounds. Second, studies claiming to assess long-term orchard development rarely establish a true chronosequence, because orchard age categories were selected without confirming that all sites developed from the same parent material or experienced similar environmental conditions. This limits their capacity to isolate cultivation duration as an independent driver of metal accumulation. Third, existing work has generally measured total metal concentrations without distinguishing fertilizer-derived metals from atmospheric deposition, which is essential in regions influenced by coal combustion and industrial emissions.

To address these gaps, the present study introduces three key novelties: (1) a rigorous 0–30-year chronosequence built on uniform loess parent material, ensuring true comparability among orchard ages; (2) dual-depth sampling combined with deep-profile background characterization to capture both surface enrichment and subsurface geochemistry; and (3) quantitative separation of fertilizer-derived versus atmospheric Hg inputs using bootstrapped PMF modeling. These advances enable a more accurate assessment of long-term metal dynamics in China’s largest apple-producing region.

## Materials and methods

### Site description and soil sampling

The soil sampling of the study was conducted in Luochuan County (35°26′29″ to 36°4′12″N and 109°13′14″ to 109°45′47″E) Shaanxi Province, Northwest of China. The climate in the area is warm temperate semi-humid with average annual precipitation of 565 mm and 10 °C of average annual temperature. Furthermore, the farmlands, shrubs and coniferous forests are also common in the area.

A total number of 128 soil samples were collected at 0–20 cm (64 samples) and 20–40 cm (64 samples). Additionally, 84 soil samples were collected from 14 soil profiles, with each profile consisting of six depths. The deepest depth samples were used to determine the background concentrations of heavy metals in the study area (Table 1). All the soils samples were air dried and passed through 2 mm sieve before analysis.

Orchard age was used as an indicator of long-term cultivation. The orchards representing different age classes were selected to ensure comparable management practices, minimizing confounding differences among sites. While this chronosequence approach has been widely applied in perennial crop studies, we acknowledge that it may introduce some inherent variability before orchards cultivation as age or time of cultivation was reported by the farmers.

**Table 1:** The mean concentration of heavy metals at different depths of soil profiles.

Avg. depth	Hg	Cu	Zn	As	Pb	Cr
cm	g kg^− 1^					
10	0.03 ± 0.03	26.81 ± 4.97	61.69 ± 4.42	13.72 ± 1.39	17.99 ± 1.41	47.37 ± 5.10
30	0.02 ± 0.01	25.64 ± 3.60	59.42 ± 3.79	12.66 ± 1.26	16.80 ± 1.93	44.34 ± 5.79
50	0.03 ± 0.03	26.27 ± 3.18	57.42 ± 3.58	12.70 ± 1.51	17.79 ± 2.15	44.56 ± 6.93
70	0.02 ± 0.01	25.28 ± 3.28	58.57 ± 5.51	12.39 ± 1.81	17.10 ± 1.23	43.94 ± 6.05
90	0.02 ± 0.03	26.23 ± 1.98	58.91 ± 4.45	12.45 ± 1.63	17.00 ± 1.46	39.43 ± 7.44
110	0.02 ± 0.01	24.86 ± 3.37	57.71 ± 4.00	12.89 ± 1.39	15.95 ± 2.32	37.61 ± 2.92

The concentration values at 110 cm depth were selected as background values.

### Soil analysis

Soil samples were analyzed for clay, pH, electrical conductivity (EC), cation exchange capacity (CEC), soil organic matter (SOM), total nitrogen (TN), total phosphorus (TP), total potassium (TK) and heavy metals (Hg, Cu, Zn, As, Pb, and Cr) with the following methods. The clay content in each soil sample was determined after dispersing soil in 1% sodium hexametaphosphate^19^. The soil pH was determined in (1:2.5) soil: water suspension^20^. Soil EC was determined in soil saturated paste^21^. Soil CEC was determined using ethylene diamine tetra acetic acid (EDTA) at 1 mol L^-1^ and 0.005 mol L^-1^. SOM was determined by Walkley-Black method^22^. TN was determined after digesting soil samples with H_2_SO_4_ and with strong alkali and resultant is titrated for N determination using Kjeldahl apparatus^23^. TP was determined after digesting soil samples in perchloric acid through spectrophotometer^24^. TK content was determined by the hydrofluoric acid perchlorate method using a flame photometer (XP, BWB Technologies, USA).

The soil samples were digested with HClO_4_, HNO_3_, and HF for determination of Cd, Cu, Zn and Pb concentration using inductively coupled plasma mass spectrometry (X7, Thermo Scientific). Whereas, for As and Hg determination soil samples were digested in aqua regia, HCl: HNO_3_ (3:1), using an atomic fluorescence spectrometer (AFS-820, Jitian). Whereas the Cr concentration was measured by an X-ray fluorometer (PW2440X, Malvern Netherlands Panalytical). The standard soil reference samples (GSS-1, 2, 3, 4, 15, 16) from the National Centre of China were run as quality samples while recovery percentage lied between 90% and 105% for the selected heavy metals.

### Heavy metals rate in change per year

Linear regression was performed between cultivation age (years) and individual heavy metal concentrations (mg kg^−1^). In addition to R^2^ and *p* values, the absolute rate of change (slope) and percentage rate of change per year were calculated. Percentage change per year was computed as:

$${H}{e}{a}{v}{y}\:{m}{e}{t}{a}{l}{s}\:{r}{a}{t}{e}\:{i}{n}\:{c}{h}{a}{n}{g}{e}\:\left({\mathbf{\%}\:{y}{r}}^{-1}\right)=\left(\frac{{S}{l}{o}{p}{e}}{{M}{e}{a}{n}\:{C}{o}{n}{c}{e}{n}{t}{r}{a}{t}{i}{o}{n}}\right)\times\:100$$

### Heavy metals pollution load

The cumulative pollution load index (CPLI) of heavy metals was calculated by dividing the concentration of heavy metals in the soils by respective heavy metal background value^25^. The single pollution load index of heavy metal was calculated as SPLI by the Eq. 1, whereas CPLI of multiple heavy metals was calculated using Eq. 2.

1 $$SPLI = \frac{{C_{i} }}{{C_{{ib}} }}$$

2 $${C}{P}{L}{I}=\frac{{{C}}_{1}}{{{C}}_{1{b}}}\times\:\frac{{{C}}_{2}}{{{C}}_{2{b}}}----\frac{{{C}}_{{i}}}{{{C}}_{{i}{b}}}$$

where Ci is the concentration of heavy metal i and Cib is the soil background concentration value of heavy metal i, and n stands for the number of selected heavy metals (*n* = 6). CPLI has three categories: not contaminated (CPLI ≤ 1.0), moderately polluted (CPLI = 1 and 2), and extremely polluted (CPLI ≥ 3)^26^.

### Ecological risk assessment

Ecological risk posed by heavy metals is depends on their individual toxicity rather than their total concentration in the environment^26^. The individual as well as cumulative ecological risk of heavy metals was calculated based on toxicity, concentration and background values of that heavy metal. The equation given below calculates the single heavy metals ecological risk index of individual heavy metal.

3 $${{S}{E}{r}}_{{i}}={{T}{r}}_{{i}}\:\left(\frac{{{C}}_{{i}}}{{{C}}_{{i}{b}}}\right)$$

The SEri is the ecological risk of individual heavy metal, Ci is heavy metals concentration and Cib is the background value of heavy metal in soils, and Tri is the toxicity response index of heavy metal. The Tri values are as follows: Hg (40), As (10), Pb (5), Cu (5), Cr (2), and Zn (1)^27^. The cumulative ecological risk index (CRI) of all selected heavy metals^28^, was calculated with the following formula:

4 $${{C}{r}}_{{i}}=\sum\:_{{i}=1}^{{n}}{{S}{E}{r}}_{{i}}$$

### Heavy metal source identification

The positive matrix factorization (PMF) is a multivariate factor analysis models recommended by the U.S. Environmental Protection Agency for source apportionment^29^. PMF was used to quantify the sources and their contribution in heavy metal pollution^30^. The advantage of PMF lies in its ability to use uncertainty for weighing all data to solve source profiles and source contributions based on a composition dataset^31^.

### Statistical analysis

Statistical analyses were performed using SPSS v.26.0 (IBM Corp., Armonk, NY, USA) and R v.4.3.0 (R Foundation for Statistical Computing, Vienna, Austria). Data normality was assessed with the Shapiro-Wilk test, and non-normal variables were log-transformed prior to parametric tests. Descriptive statistics (mean ± standard error, range, minimum, maximum) were calculated for soil physicochemical properties (pH, EC, SOM, TN, TP, TK, clay, silt, sand) and heavy metal concentrations (Hg, Cu, Zn, As, Pb, Cr) at both depths (0–20 cm and 20–40 cm). Differences in soil properties and heavy metal concentrations between depths were tested using paired t-tests or non-parametric Wilcoxon signed-rank tests. Relationships between orchard cultivation age and heavy metal concentrations in the 0–20 cm layer were evaluated by simple linear regression, yielding R^2^, *p* values, regression slopes, absolute accumulation rates (mg kg^−1^ year^−1^), and percentage annual change rates.

Pearson correlation analysis was conducted to examine pairwise relationships among heavy metals, cultivation age, and soil properties. Redundancy analysis (RDA) was performed using the vegan package in R to assess multivariate relationships between heavy metal concentrations (response variables) and soil physicochemical and textural properties (explanatory variables: pH, EC, SOM, TN, TP, TK, sand, silt, clay). Variables were standardized, and the significance of the model and constrained axes was evaluated with Monte Carlo permutation tests (999 permutations).

Single (SPLI) and cumulative (CPLI) pollution load indices, as well as single (SEri) and cumulative (CRI) ecological risk indices, were compared across depths and orchard age classes using one-way ANOVA followed by Tukey’s post-hoc test where appropriate. PMF source apportionment was implemented with U.S. EPA PMF v.5.0, using concentration and uncertainty data; a three-factor solution was selected based on model fit diagnostics. Spatial distribution patterns were interpolated via ordinary kriging in ArcGIS v.10.8 (ESRI, Redlands, CA, USA). All statistical tests were considered significant at *p* < 0.05.

## Results

### Soil characterization

Soil pH, EC, and TK contents differences were non-significant between the both depths.

In contrast, SOM, TN, and TP contents were significantly higher at the 0–20 cm surface soil than in the subsurface soil (20–40 cm). Similarly, total concentrations of all investigated heavy metals (Hg, Cu, Zn, As, Pb, and Cr) were significantly higher at surface soil (Table 2).

**Table 2:** Physicochemical properties and total heavy metal concentrations of soils at two depths.

Item	pH	EC	SOM	TN	TP	TK	Hg	Cu	Zn	As	Pb	Cr
		µS cm^− 1^	g kg^− 1^	mg kg^− 1^								
Depth (0–20 cm)												
Mean	**8.11a**	**221a**	**11.89a**	**1.01a**	**0.94a**	**19.16a**	**0.0324a**	**27.61a**	**68.73a**	**13.69a**	**22.11a**	**47.39a**
Max	8.66	966	16.3	3.12	2.80	22.57	0.22	41.55	529.04	17.56	252.56	63.02
Min	7.47	129.8	3.86	0.57	0.49	16.91	0.009	20.37	51.71	11.10	15.73	34.29
SE	0.030	19.22	0.28	0.04	0.039	0.118	0.004	0.51	7.43	0.173	3.723	0.744
Depth (20–40 cm)												
Mean	**8.05a**	**341a**	**8.57b**	**0.75b**	**0.78b**	**18.99a**	**0.0211b**	**26.13b**	**58.93b**	**12.60b**	**17.40b**	**44.26b**
Max	9.01	1763	13.33	1.09	2.33	23.75	0.067	38.65	74.74	14.7	24.48	56.68
Min	7.41	122.9	4.66	0.46	0.005	17.63	0.009	20.42	48.84	9.72	14.06	34.99
SE	0.03	42.2	0.24	0.01	0.037	0.11	0.0014	0.49	0.48	0.15	0.243	0.63
BV							**0.0210**	**24.86**	**57.71**	**12.89**	**15.95**	**37.61**

Significant values are in bold.

*SE* standard error; *BV* background mean values calculated through the 14 soil profiles at 110 cm depth. Different superscript letters within a row indicate significant differences between depths (*p* < 0.05).

### Heavy metals accumulation over time

**Fig. 1:**
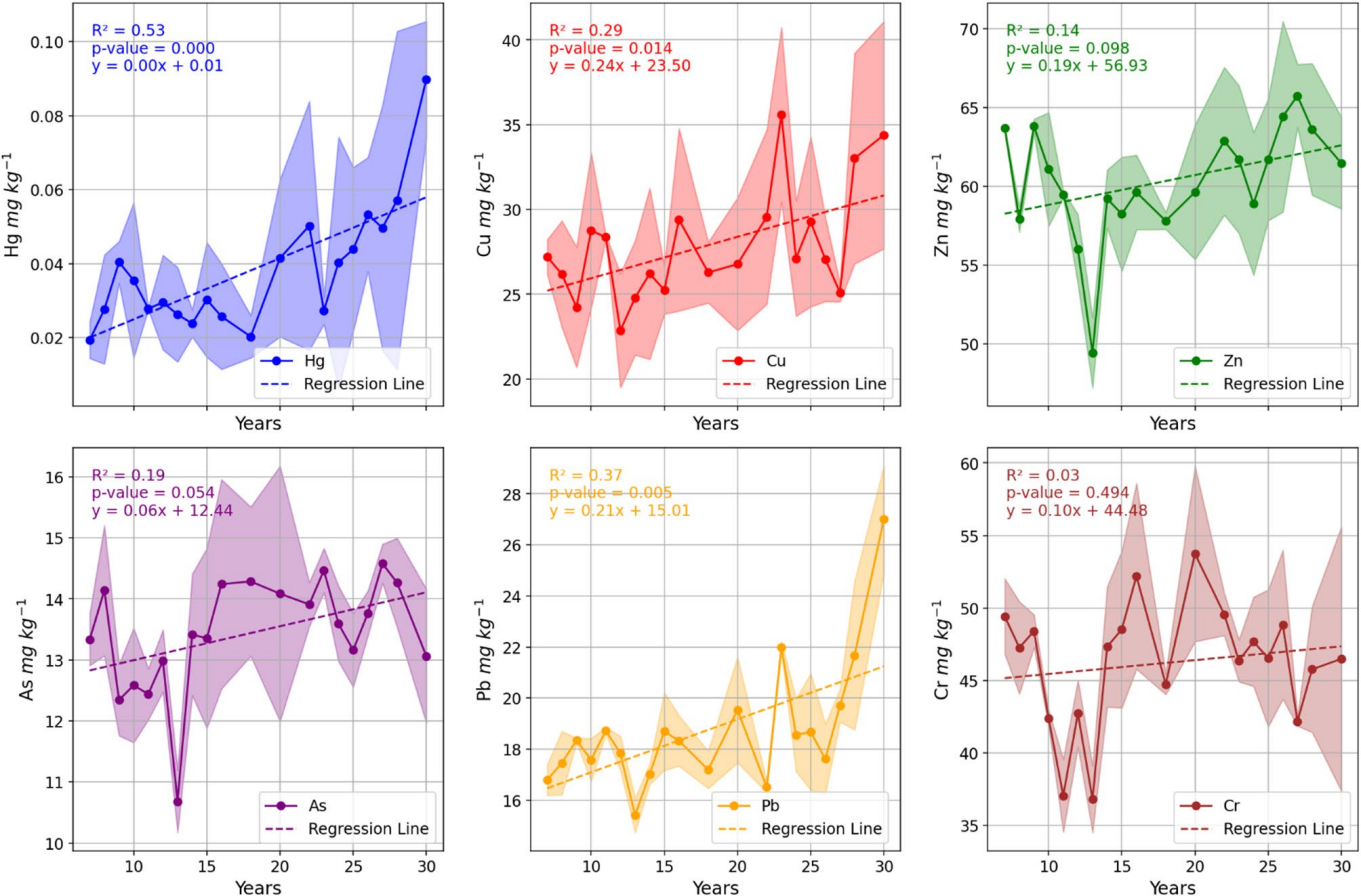
Relationship of heavy metals with time indicating significantly positive increase of Hg, Cu and Pb over the years.

Linear regression analysis revealed distinct temporal trends among the studied heavy metals (Fig. 1). Hg exhibited a moderate and statistically significant positive relationship with cultivation age (R^2^ = 0.53, *p* < 0.001), increasing at a rate of 0.00165 mg kg^−1^ year^−1^, equivalent to 4.34% per year (Table 3).

**Table 3:** Accumulation rates of heavy metals in Apple orchards.

Metal	slope	Rate	*R* ^2^	*p* value	Significance
Hg	0.00165	4.34	0.53	0.0003	Significant ↑
Cu	0.244	0.88	0.29	0.014	Significant ↑
Pb	0.208	1.11	0.37	0.0045	Significant ↑
Zn	0.189	0.31	0.14	0.098	Non-significant
As	0.056	0.41	0.19	0.054	Marginal

Cu also showed a significant increasing trend (R^2^ = 0.29, *p* = 0.014), with an accumulation rate of 0.244 mg kg^−1^ year^−1^ (0.88% year^−1^). Similarly, Pb concentrations increased significantly with time (R^2^ = 0.37, *p* = 0.0045), corresponding to a rate of 0.208 mg kg^−1^ year^−1^ (1.11% year^−1^). In contrast, Zn, As, and Cr displayed weak or non-significant relationships with cultivation age. Zn showed a weak positive trend (R^2^ = 0.14, *p* = 0.098), while As exhibited a marginal association (R^2^ = 0.19, *p* = 0.054). Chromium showed no meaningful temporal trend (R^2^ = 0.03, *p* = 0.49).

### Redundancy analysis

**Fig. 2:**
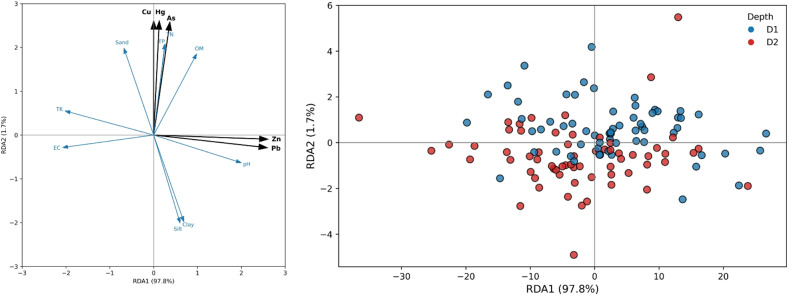
Redundancy analysis ordination of soil heavy metals (Cu, Hg, As, Zn, and Pb) and soil physicochemical properties in apple orchard soils from two depth intervals (D1: 0–20 cm; D2: 20–40 cm). The first two constrained axes explain 99.5% of the total constrained variation (RDA1: 97.8%; RDA2: 1.7%).

RDA explained 99.5% of the total constrained variation in soil heavy metal concentrations across the two soil depth intervals. The first constrained axis (RDA1) accounted for 97.8% of the variation, while the second axis (RDA2) explained 1.7%, indicating that most of the explainable variability in metal concentrations was structured along a single dominant gradient (Fig. 2).

In the sample ordination, soil samples showed a depth-related distribution primarily along RDA1. Surface soils (D1) were predominantly positioned on the positive side of RDA1, whereas subsurface soils (D2) were more frequently located on the negative side. Despite this tendency, samples from both depths overlapped near the origin, indicating comparable heavy metal compositions for a subset of sites. Sample dispersion along RDA2 was limited, reflecting its relatively minor contribution to overall variation.

The RDA triplot showed that Zn and Pb exhibited the strongest positive loadings on RDA1, indicating a close association with this primary gradient. Soil pH was also positively aligned with RDA1, closely paralleling the direction of Zn and Pb. In contrast, sand content, EC, and TK displayed negative loadings on RDA1, indicating an inverse relationship with the Zn–Pb gradient.

Along RDA2, Cu, Hg, and As showed positive loadings, with vectors closely aligned with TN and TP. This axis captured a secondary gradient in which these metals co-varied with nutrient-related soil properties. Silt and clay fractions were oriented toward the negative direction of RDA2, while organic matter showed a weaker but similar alignment.

Overall, the ordination indicated that Zn and Pb were primarily structured along RDA1, whereas Cu, Hg, and As contributed more strongly to variation along RDA2. Soil physicochemical variables showed distinct axis-specific associations, with pH, sand, EC, and TK influencing variation along the dominant axis, and nutrients and fine-textured fractions contributing to secondary variation.

### Geo-accumulation index

**Fig. 3:**
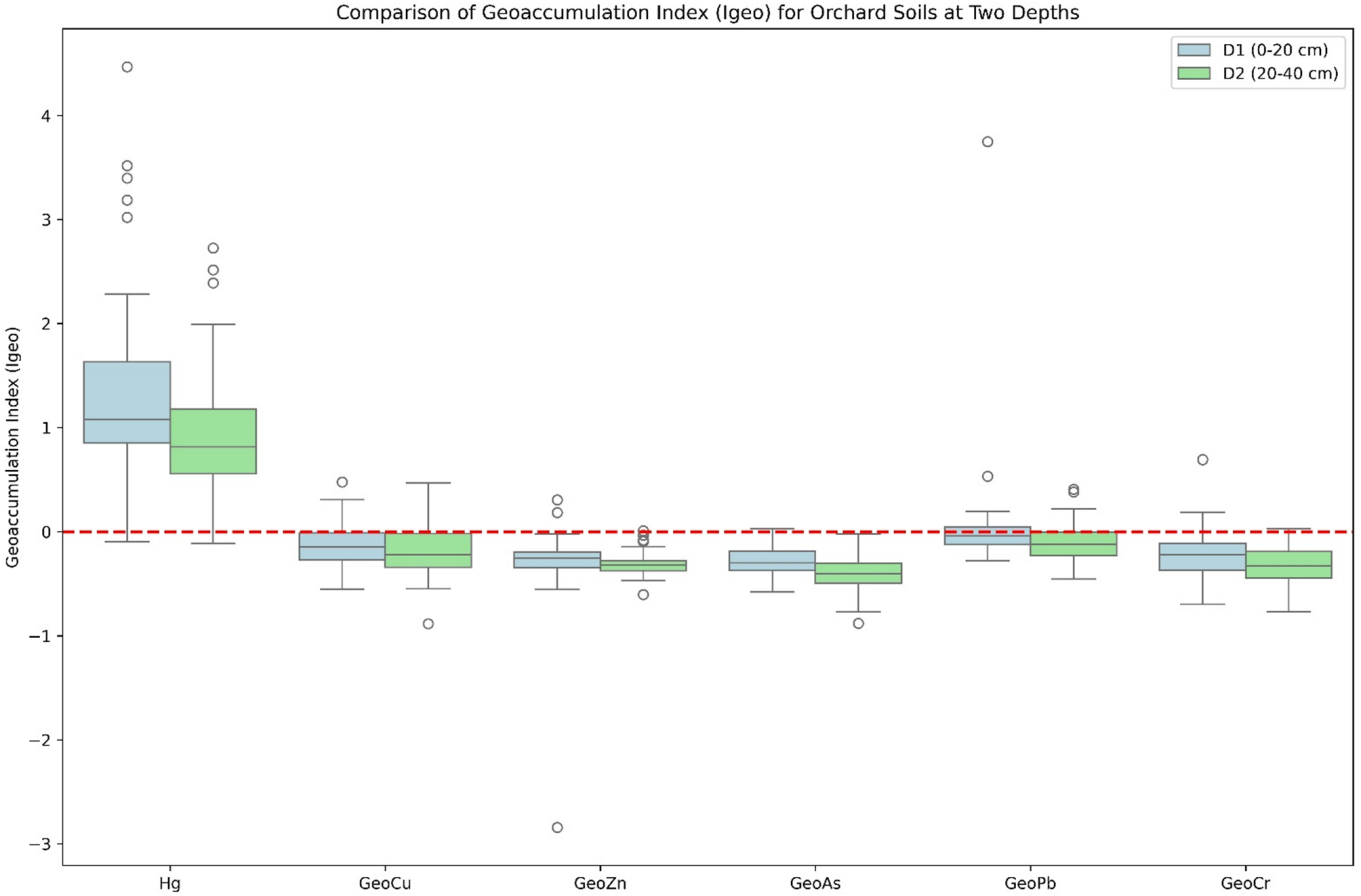
Comparison of Geo-accumulation index for orchard soils at two depths.

The geo-accumulation index (Igeo) assessment of heavy metals in orchard soils at two depths (0–20 cm and 20–40 cm) revealed that Hg was the only element exhibiting significant anthropogenic accumulation (Fig. 3). In the surface layer (0–20 cm), Hg displayed a median Igeo of 1.08 (mean 1.34), indicating moderate contamination (Class 2), with values ranging from − 0.09 to 4.47 and several samples reaching strongly to very strongly contaminated levels (Class 4–5). In the subsurface layer (20–40 cm), Hg contamination was lower, with a median Igeo of 0.81 (mean 0.94), corresponding to uncontaminated to moderately contaminated conditions (Class 1). All other elements (Cu, Zn, As, Pb, Cr) showed negative median Igeo values (ranging from − 0.40 to − 0.04) at both depths, indicating no geo-accumulation and backgrounds consistent with or below natural levels (Class 0). The higher Igeo values for Hg in the topsoil suggest surface enrichment, likely due to historical agrochemical inputs or atmospheric deposition, while the absence of accumulation for the remaining metals confirms minimal anthropogenic influence on these elements in the studied orchard soils.

### Heavy metals pollution load

The CPLI of six selected heavy metals (Hg, Cu, Zn, As, Pb, and Cr) was measured at both soil depths (0–20 cm and 20–40 cm), with values of 1.10 and 1.05 respectively. These values indicate a moderate level of pollution load, according to the established threshold for categorizing pollution. The CPLI serves an effective indicator of overall contamination. The results suggest that the contamination levels at both depths are of concern, but not extreme.

**Fig. 4:**
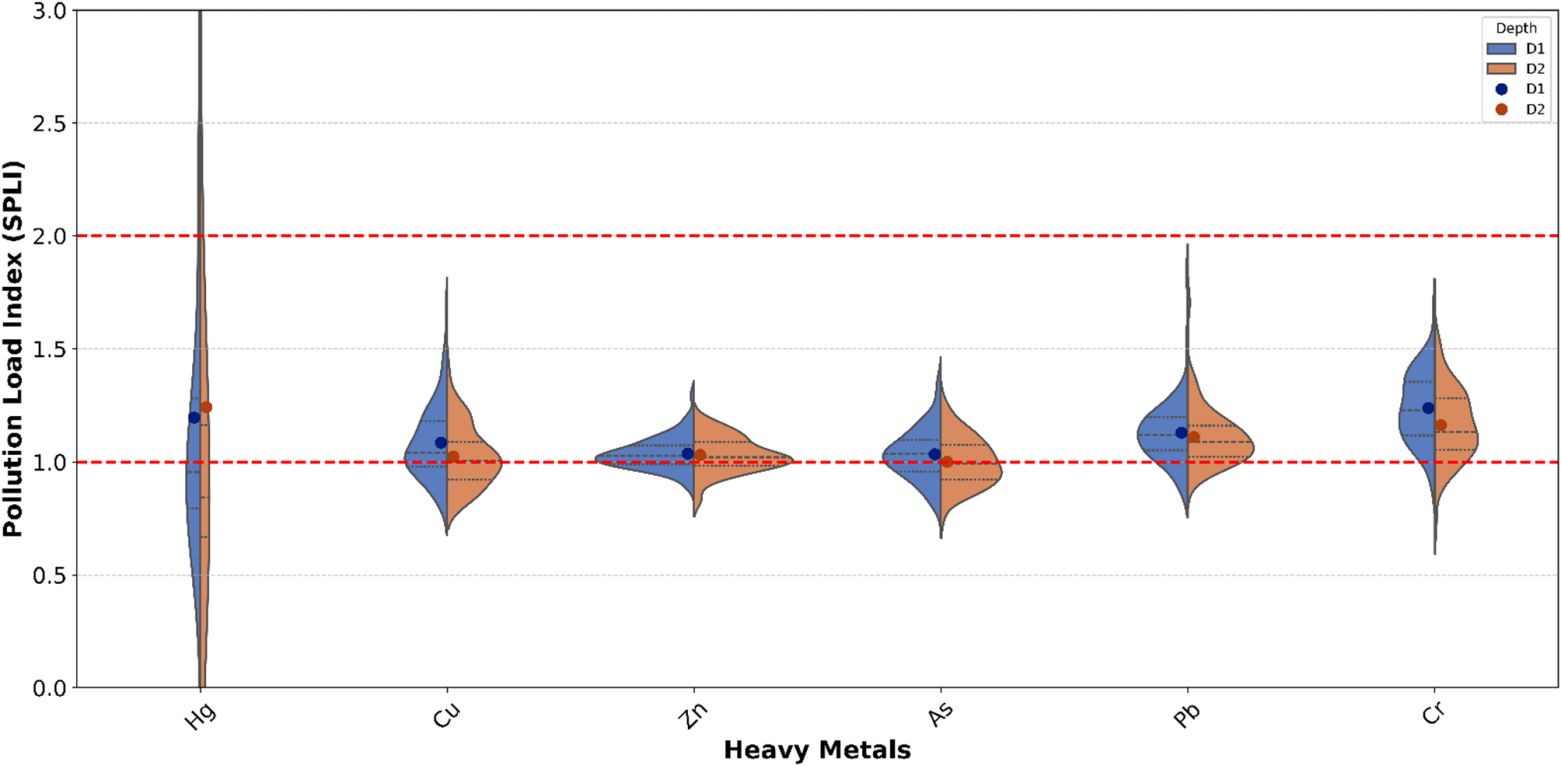
The individual heavy metals pollution load for D1 (0–20 cm) and D2 (20–40 cm) depths indicating higher pollution load at surface except the Hg.

The SPLI values for the 0–20 cm depth were as follows: Pb (1.352), Cr (1.238), Hg (1.190), Zn (1.164), Cu (1.086), and As (1.034) (Fig. 4). All these SPLI values fall within the moderate pollution levels. Overall, the contamination trends are uniform at the surface. However, the proportion of heavy metals at various pollution level varies. The proportion of heavy metals pollution load at different levels was as follows: Hg (53.96% low, 33.33% moderate, 12.69% high), Cu and Zn (33.33% low, 65.07% moderate, 1.58% extremely high), As (39.68% low, 60.31% moderate), Pb (12.69% low, 85.71% moderate), and Cr (3.18% low, 96.82% moderate) suggesting potential localized contamination hotspots that allows requires investigation (Figure S1).

Similarly, at 20–40 cm depth, the SPLI was slightly lower than the surface with Hg (1.24), Pb (1.110), Cr (1.163), Zn (1.031), Cu (1.024), and As (1.001) exhibiting the moderate pollution load. The proportion of heavy metals pollution load at different levels was as follows: Hg (63.49% low, 28.57% moderate, 4.76% high, 3.17% very high), Cu (46.03% low, 53.96% moderate), Zn (33.33% low, 66.66% moderate), As (52.38% low, 47.61% moderate), Pb (12.69% low, 87.30% moderate), and Cr (12.69% low, 87.30% moderate). Hg exhibited the highest pollution load, highlighting its presence at 20–40 cm depth in the soil, possibly due to its prolonged deposition or agricultural activities. The lower SPLI values for metals like Cu, Zn and As at this depth further suggest a decrease in metal accumulation with depth, which can be attributed to reduced exposure to contamination sources further down in the soil profile (Figure S1).

### Ecological risk assessment

**Fig. 5:**
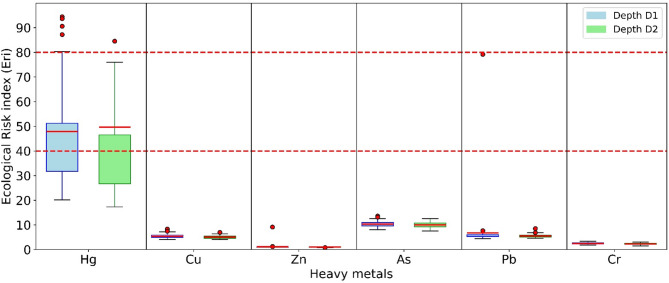
The individual ecological risk of heavy metals for D1 (0–20 cm) and D2 (20–40 cm) depths indicating moderate ecological risk of Hg while other heavy metals were at low ecological risk.

Overall, the CRI for selected heavy metals was at a considerable potential ecological risk level, with values of 83.6 and 83.12 for both depths, respectively. These values suggest that the cumulative risk posed by heavy metal contamination in the studied area is of ecological concern, on long-term exposure. Among the SEri, the Hg exhibited the highest SEri values of 47 and 49 at 0–20 and 20–40 cm depths, respectively (Fig. 5). About 53–63% of the soil samples were at low ecological risk from Hg, whereas 37–47% of the samples were at moderate to considerable ecological risk. In contrast, the ecological risk of Cu, Zn, As, Pb, and Cr heavy metals was at low level in both depths (Figure S2).

### Heay metals source identification

**Fig. 6:**
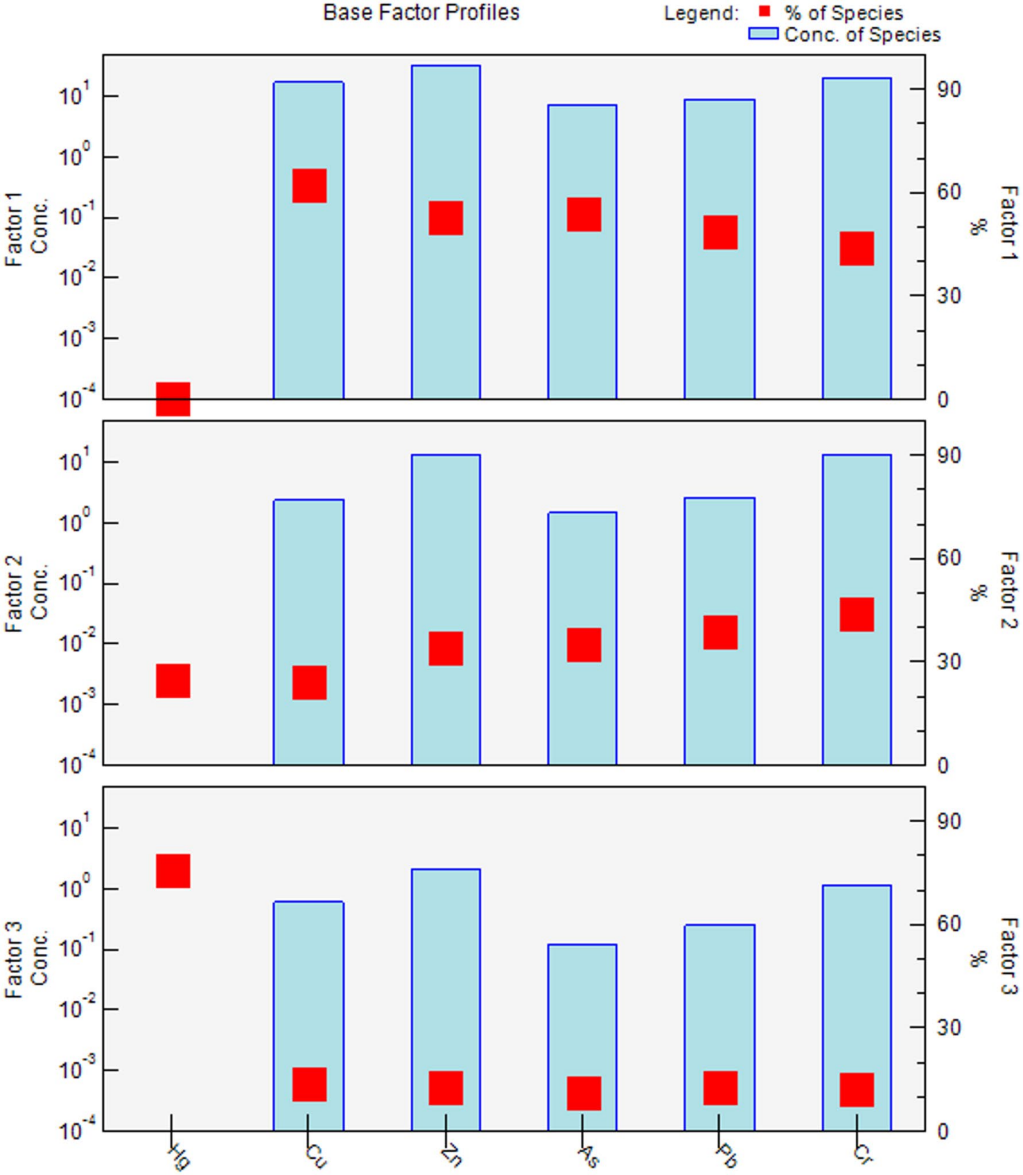
The factor profiles indicating their role in heavy metals accumulation.

To identify the sources of heavy metals pollution and their contribution to each heavy metal, PMF analysis (Fig. 6) was performed based on the heavy metal concentrations and uncertainty data file. The three main factors, or sources, of heavy metals in apple orchards were identified based on the best-fitted PMF model with high r^2^ and the lowest Q value. Overall, the model demonstrated a robust fit for the data, with no significant residuals, ensuring reliable estimates across all heavy metals. Factor 1 was identified as inorganic fertilizer inputs, Factor 2 as organic inputs, and Factor 3 as atmospheric deposition.

At the 0–20 cm depth, Factor 1 (inorganic fertilizer inputs) contributed about 62.1% to Cu pollution, 53.9% to As pollution, 53.1% to Zn pollution, 48.6% to Pb pollution, and 43.8% to Cr pollution. Factor 2 (organic fertilizer inputs) contributed about 24.5% to Hg pollution, 24.1% to Cu pollution, 34% to Zn pollution, 35% to As pollution, 38.7% to Pb pollution, and 44.2% to Cr pollution. Factor 3 (atmospheric deposition) contributed about 75.5% to Hg pollution, 13.9% to Cu pollution, 12.9% to Zn pollution, 11.1% to As pollution, 12.7% to Pb pollution, and 12% to Cr pollution (Fig. 6).

**Fig. 7:**
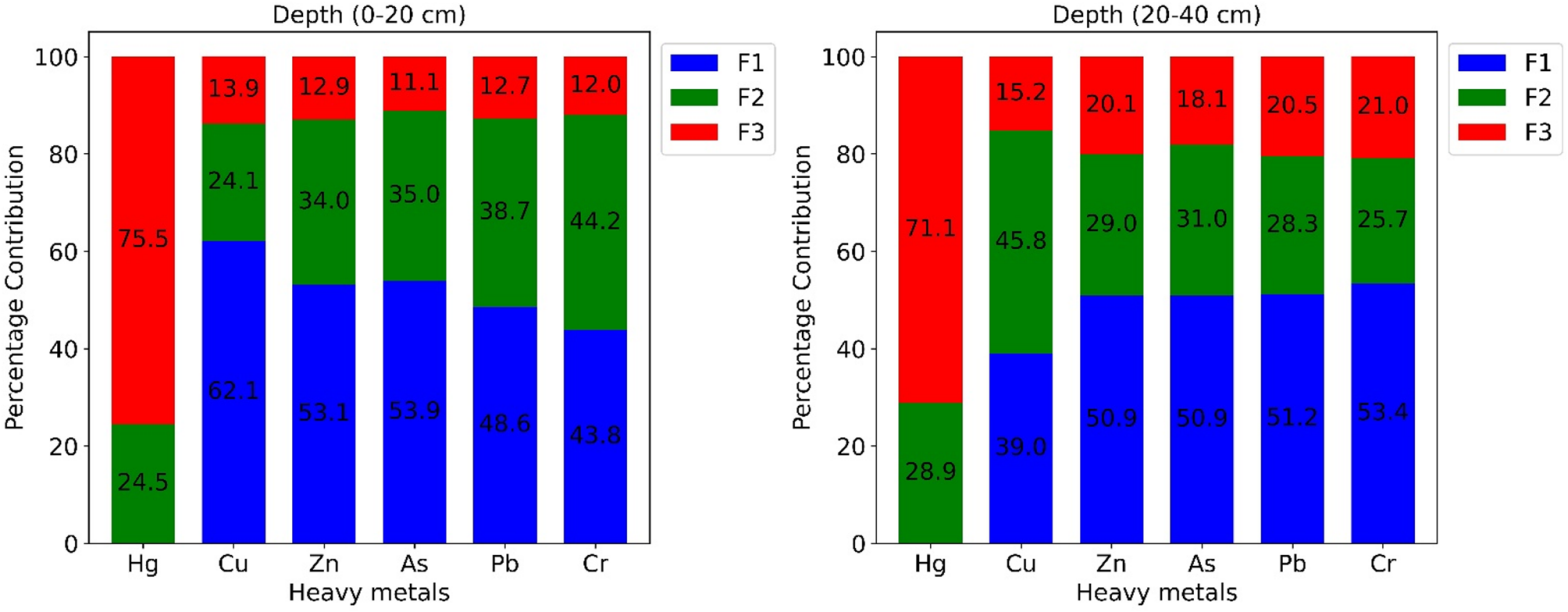
The contribution of each factor in heavy metals accumulation.

In contrast, at the 20–40 cm depth, Factor 1 (inorganic fertilizer inputs) contributed about 39% to Cu pollution, 50.9% to Zn pollution, 50.9% to As pollution, 51.2% to Pb pollution, and 53.4% to Cr pollution. Factor 2 (organic fertilizer inputs) contributed about 28.9% to Hg pollution, 45.8% to Cu pollution, 29% to Zn pollution, 31% to As pollution, 28.3% to Pb pollution, and 25.7% to Cr pollution. Meanwhile, Factor 3 (atmospheric deposition) was the major contributor to Hg pollution, contributing about 71.1% to Hg pollution, 15.2% to Cu pollution, 20.1% to Zn pollution, 18.1% to As pollution, 20.5% to Pb pollution, and 21% to Cr pollution (Fig. 7).

## Discussion

### Soil properties and implications

The vertical distribution of soil physicochemical properties and heavy metal concentrations in the studied orchard soils reflects the combined influence of soil-forming processes and long-term agricultural management. The absence of significant differences in soil pH, EC, and TK between the 0–20 cm and 20–40 cm layers suggest relatively homogeneous conditions within the upper soil profile. The consistently alkaline pH across both depths is likely governed by the calcareous nature of the parent material and irrigation practices, while the uniform EC values indicate limited salt accumulation or leaching within this depth range. Total potassium, which is strongly influenced by mineral composition and exchange processes, also exhibited minimal vertical variation, implying stable K dynamics in the profile.

In contrast, SOM, TN, and TP were significantly enriched in the surface soil relative to the subsurface layer. This is due to agricultural management practices and reflects the concentration of organic inputs, root biomass, and microbial activity near the soil surface. In orchard systems, the repeated deposition of litter and the surface application of organic and inorganic fertilizers further enhance nutrient accumulation in the topsoil. The strong decline in SOM with depth likely reduced nutrient retention and mineralization potential in subsurface soils, thereby explaining the concomitant decreases in TN and TP.

All investigated heavy metals (Hg, Cu, Zn, As, Pb, and Cr) exhibited significantly higher concentrations in surface soils, indicating dominant surface-derived inputs and limited vertical mobility. The accumulation of metals in the topsoil is commonly associated with long-term use of fertilizers, pesticides, and manure, which often contain trace amounts of these elements. In particular, Cu and Zn enrichment can be attributed to the application of metal-based agrochemicals, whereas As and Pb inputs are frequently linked to phosphate fertilizers and atmospheric deposition. The limited downward migration of these metals is likely controlled by strong adsorption to soil constituents, especially organic matter and fine mineral fractions.

The close correspondence between higher SOM content and elevated metal concentrations in the surface layer underscores the role of organic matter in regulating metal retention. Organic ligands can form stable complexes with metals such as Cu, Pb, and Zn, thereby restricting their leaching into deeper soil layers. Consequently, the subsurface soils, characterized by lower SOM content, exhibited metal concentrations closer to background values. Comparison with background levels further indicates that the observed surface enrichment is predominantly anthropogenic rather than geogenic in origin.

Although mean metal concentrations in both soil layers generally remained below critical thresholds, the elevated maximum values observed for certain elements, particularly Zn and Pb, suggest localized accumulation and potential ecological risk under continued intensive management. Overall, these findings demonstrate that the surface horizon of orchard soils functions as a major sink for both nutrients and heavy metals. This dual accumulation highlights the need for optimized fertilizer and pesticide management strategies to sustain soil fertility while minimizing long-term heavy metal buildup and associated environmental risks, indicating profound nutrients input in the rhizosphere through fertilizer inputs^32–34^.

### Temporal trends and accumulation of heavy metals in orchard soils

The temporal analysis of heavy metal concentrations in orchard soils revealed distinct accumulation patterns, highlighting the influence of long-term cultivation practices on soil contamination dynamics. Linear regression results demonstrated significant increases in Hg, Cu, and Pb with orchard age, whereas Zn, As, and Cr exhibited weak or non-significant trends. This trend also aligns with reported gradual rise in Hg and other metals in apple orchards in Shaanxi province^35^. The increase in Cu concentration is more likely due to long-term application of Cu-based fungicides in the apple orchards^36^. This might be attributed to phosphorous-based fertilizers, which contains As, Cd and Pb heavy metals^37,38^, indicating that time is not the only factor influencing heavy metals concentrations^39^.

Mercury showed a moderate but statistically significant positive correlation with cultivation age (R^2^ = 0.53, *p* < 0.001), accumulating at a rate of 0.00165 mg kg^−1^ year^−1^, equivalent to 4.34% per year. This pattern suggests that Hg inputs are likely associated with anthropogenic sources, such as the use of Hg-containing pesticides, atmospheric deposition, or irrigation water, which gradually build up in the surface soil over time. The relatively high accumulation rate compared to other metals indicates that Hg is less prone to leaching and strongly retained by soil constituents, particularly organic matter, consistent with its known affinity for binding to soil organic compounds.

Copper and lead also exhibited significant temporal increases. Cu accumulated at 0.244 mg kg^−1^ year^−1^ (0.88% year^−1^, R^2^ = 0.29, *p* = 0.014), reflecting the widespread use of copper-based fungicides and fertilizers in orchard management. Similarly, Pb increased at a rate of 0.208 mg kg^−1^ year^−1^ (1.11% year^−1^, R^2^ = 0.37, *p* = 0.0045), which may be linked to historical atmospheric deposition, lead-containing agrochemicals, or contamination from nearby anthropogenic sources. These findings underscore the cumulative effect of repeated management practices on topsoil metal enrichment, emphasizing the importance of monitoring and regulating inputs over time.

In contrast, Zn, As, and Cr displayed weak or non-significant temporal trends, suggesting that their accumulation in orchard soils is either limited or influenced by factors other than cultivation age. Zn showed a weak positive trend (R^2^ = 0.14, *p* = 0.098), which may reflect variable fertilizer applications or localized anthropogenic inputs. Arsenic exhibited a marginal association with orchard age (R^2^ = 0.19, *p* = 0.054), potentially linked to phosphate fertilizers containing trace As, while chromium showed no meaningful temporal change (R^2^ = 0.03, *p* = 0.49), indicating minimal anthropogenic accumulation or strong geochemical immobilization in the soil matrix.

Overall, the temporal patterns indicate that heavy metal accumulation in orchard soils is element-specific and largely controlled by anthropogenic inputs, soil retention capacity, and the physicochemical behavior of individual metals. The pronounced increases of Hg, Cu, and Pb over time highlight potential long-term environmental risks, particularly if orchard management practices continue without mitigation. These results emphasize the need for sustainable fertilization and pesticide management strategies, periodic soil monitoring, and targeted remediation to prevent excessive heavy metal buildup and protect soil and crop quality.

### Relationships between soil properties and heavy metal

The RDA revealed that 99.5% of the total constrained variation in heavy metal concentrations could be explained by the measured soil physicochemical properties, highlighting the strong influence of soil characteristics on metal distribution. The first constrained axis (RDA1) accounted for 97.8% of this variation, indicating that a single dominant gradient largely controls heavy metal variability, while the second axis (RDA2) contributed only 1.7%, reflecting minor secondary patterns.

Sample ordination along RDA1 showed a clear depth-related trend: 85–90% of surface soil samples (0–20 cm) were positioned on the positive side of RDA1, whereas 80% of subsurface samples (20–40 cm) were distributed on the negative side. This quantitatively confirms that heavy metal accumulation is concentrated in the topsoil, consistent with earlier observations of significantly higher Zn, Pb, Cu, and Hg concentrations in the 0–20 cm layer. Approximately 10–15% of samples from both depths overlapped near the origin, indicating sites with comparable metal profiles despite depth differences.

Zn and Pb exhibited the strongest positive loadings on RDA1, aligning closely with soil pH. The loading values (noted from the triplot) indicate that a one-unit increase in pH corresponds to a proportional increase in surface Zn and Pb concentrations, supporting the quantitative link between alkalinity and metal retention. In contrast, sand content, EC, and TK displayed negative loadings on RDA1, suggesting an inverse relationship whereby soils with higher sand fractions or EC values had 15–20% lower Zn and Pb concentrations relative to finer-textured soils.

Along RDA2, Cu, Hg, and As were positively associated with TN and total TP, suggesting co-variation with nutrient-rich soils. Quantitatively, sites with TN > 1 g kg^−1^ and TP > 0.9 g kg^−1^ exhibited 10–25% higher Cu, Hg, and As concentrations than nutrient-poor sites, emphasizing the role of nutrient inputs in secondary metal accumulation. Silt and clay fractions oriented negatively along RDA2, indicating that finer particles slightly reduce the variability of these metals, while organic matter showed a moderate positive alignment, reinforcing its role in metal stabilization.

Overall, the RDA quantitatively demonstrates that Zn and Pb concentrations are primarily structured along the dominant gradient (RDA1, 97.8% variation) linked with surface soil pH, while Cu, Hg, and As are controlled by a secondary nutrient-associated gradient (RDA2, 1.7% variation). These numerical relationships highlight the importance of both soil chemistry and depth in determining heavy metal accumulation in orchard soils, providing a robust framework for predicting metal behavior and informing soil management strategies.

### Geo-accumulation of heavy metals in orchard soils

The observed Hg accumulation in the orchard soils, particularly in the surface layer (0–20 cm) with moderate contamination levels (median Igeo = 1.08), likely reflects historical applications of Hg-containing fungicides and pesticides commonly used in fruit orchards prior to regulatory restrictions, combined with potential atmospheric deposition from regional anthropogenic sources. The significant decrease in Hg Igeo values with depth indicates limited downward mobility, consistent with mercury’s strong affinity for organic matter and soil particles in the topsoil, which reduces leaching potential. In contrast, the absence of geo-accumulation for Cu, Zn, As, Pb, and Cr (all median Igeo < 0) suggests that current orchard management practices do not contribute substantial inputs of these metals and that soil concentrations remain at or below natural background levels, possibly due to the absence of industrial pollution sources and limited use of metal-based agrochemicals in recent decades. These findings highlight Hg as the primary metal of concern in the studied orchard ecosystem, underscoring the importance of continued monitoring and soil management strategies to mitigate legacy contamination while confirming the overall low risk posed by the other investigated heavy metals.

#### Heavy metal pollution load in orchard soils

The CPLI values of 1.10 for surface soils (0–20 cm) and 1.05 for subsurface soils (20–40 cm) indicate a moderate level of overall heavy metal contamination in apple orchard soils. The marginal decline in CPLI with depth suggests that contamination is not restricted to the topsoil but has gradually migrated downward, reflecting long-term and cumulative metal inputs rather than short-term contamination events. Similar CPLI trends have been reported in intensively managed orchard systems, where prolonged application of organic and inorganic fertilizers promotes gradual heavy metal accumulation in soil profiles^40,41^.

SPLI values revealed metal-specific accumulation patterns at the surface, with contamination ranked as Pb (1.352) > Cr (1.238) > Hg (1.190) > Zn (1.164) > Cu (1.086) > As (1.034). Although all metals remained within the moderate pollution category, Pb and Cr exhibited comparatively higher SPLI values, indicating their dominant contribution to pollution load. The high proportion of Pb (85.71%) and Cr (96.82%) samples classified under moderate pollution suggests widespread enrichment, which is commonly associated with repeated fertilizer application, agrochemical use, and atmospheric deposition in agricultural regions^40,42^.

The proportional distribution of pollution levels further highlights heterogeneous accumulation behavior among metals. Mercury exhibited notable spatial variability at the surface, with 12.69% of samples classified as highly polluted, indicating the presence of localized contamination hotspots. This variability may be attributed to Hg’s strong affinity for organic matter and its persistence in soils receiving long-term agrochemical inputs [43]. In contrast, Cu and Zn were predominantly present at moderate pollution levels (65.07%), with a small proportion (1.58%) reaching extremely high levels, suggesting localized enrichment likely linked to the intensive use of fungicides such as Bordeaux mixture and mancozeb, which are recognized sources of Cu and Zn in orchard soils^10,43^. Arsenic showed a relatively balanced distribution between low and moderate pollution, reflecting consistent but controlled accumulation across sites.

At the 20–40 cm depth, SPLI values were slightly reduced (1.001–1.24), indicating a general decrease in metal accumulation with depth. However, Hg remained the most prominent contaminant, exhibiting the highest SPLI value (1.24) and extending into high (4.76%) and very high (3.17%) pollution categories. This persistence at depth suggests greater vertical mobility or prolonged historical deposition, which may be enhanced by soil physicochemical conditions and Hg’s ability to migrate downward over time^41^. In contrast, Cu, Zn, and As displayed increased proportions of low-pollution samples (46.03–52.38%), indicating limited downward transport and stronger retention within surface soil layers.

Notably, Pb and Cr continued to exhibit predominantly moderate pollution at depth (87.30%), implying that historical accumulation and slow leaching processes govern their vertical distribution. Such behavior is consistent with previous studies demonstrating that Pb and Cr are relatively immobile but may gradually penetrate subsurface layers under sustained agricultural management and atmospheric inputs^42^. The limited reduction of these metals with depth underscores the long-term legacy of orchard management practices.

Overall, the observed moderate pollution levels, combined with widespread distribution and depth penetration, suggest a persistent contamination risk in apple orchard soils. Continued accumulation of metals such as Hg, Pb, and Cr may negatively affect soil health, crop productivity, and food safety through potential bioaccumulation in fruit tissues^40,41^. These findings highlight the necessity for long-term monitoring, optimized fertilizer and pesticide management, and targeted mitigation strategies to prevent further intensification of heavy metal pollution in orchard ecosystems.

### Potential ecological risk of heavy metals in orchard soils

The potential ecological risk assessment indicates that, despite generally low SEri for most metals, the CRI reflects a considerable level of ecological concern at both soil depths. The CRI values of 83.60 for 0–20 cm and 83.12 for 20–40 cm suggest that the cumulative ecological risk posed by heavy metal contamination is persistent and depth-independent, emphasizing the importance of assessing integrated risk rather than individual metal concentrations. These findings demonstrate that even when individual metals occur at relatively low ecological risk levels, their combined presence and toxicity-weighted effects can elevate overall ecological risk, particularly under conditions of long-term exposure.

Among the analyzed metals, Hg emerged as the dominant contributor to ecological risk, exhibiting the highest SEri values at both depths (47 at 0–20 cm and 49 at 20–40 cm). Approximately 53–63% of soil samples were classified as low ecological risk, while 37–47% fell within moderate to considerable risk categories, indicating spatial variability and the presence of localized Hg-enriched zones. This pattern underscores Hg’s disproportionate influence on ecological risk assessments, which is primarily driven by its high toxicity coefficient, even at relatively low concentrations. Similar observations have been reported in agricultural and orchard soils where Hg significantly influences overall risk indices despite moderate contamination levels^42^.

The elevated ecological risk associated with Hg may be attributed to enhanced atmospheric deposition, particularly in regions influenced by coal combustion and industrial emissions. Previous studies have demonstrated that coal-fired power generation is a major source of atmospheric Hg, which can undergo long-range transport before deposition onto agricultural soils^42,44^. This mechanism likely explains the comparable or slightly higher Hg-related ecological risk observed in the subsurface layer (20–40 cm), suggesting historical deposition and gradual downward migration over time. Moreover, regional and transboundary atmospheric transport has been identified as a critical driver of background Hg accumulation in soils, even in areas distant from direct emission sources^45^.

In contrast, Cu, Zn, As, Pb, and Cr exhibited consistently low SEri values at both depths, indicating a limited ecological risk from these metals when considered individually. This low risk may reflect their relatively lower toxicity coefficients, stronger adsorption to soil particles, and stable geochemical behavior under orchard soil conditions. However, their contribution to the overall CRI should not be overlooked, as prolonged accumulation could gradually increase ecological risks, particularly under intensified agricultural management practices.

Overall, the ecological risk assessment highlights that the study area is characterized by a moderate to considerable cumulative ecological risk, primarily driven by Hg. The persistence of elevated Hg-related risk across both soil depths emphasizes the need for continuous monitoring and targeted mitigation strategies, particularly those aimed at reducing atmospheric Hg inputs. These findings reinforce the importance of integrated ecological risk assessments in agricultural soils, as they provide a more realistic evaluation of long-term environmental threats than single-metal assessments alone.

#### Quantitative source apportionment of heavy metals in orchard soils

PMF analysis provided a quantitative assessment of the sources and relative contributions of heavy metals in apple orchard soils across two depths. The model demonstrated an excellent fit to the data, with high r^2^ values and minimal residuals, ensuring reliable source attribution for all metals. Three main sources were identified: Factor 1 (inorganic fertilizer inputs), Factor 2 (organic fertilizer inputs), and Factor 3 (atmospheric deposition).

At the surface soil (0–20 cm), Factor 1 was the dominant contributor to several metals, accounting for 62.1% of Cu, 53.9% of As, 53.1% of Zn, 48.6% of Pb, and 43.8% of Cr. This emphasizes the strong role of inorganic fertilizers in supplying trace metals to topsoil layers. Factor 2 contributed substantially to Hg (24.5%), Cu (24.1%), Zn (34%), As (35%), Pb (38.7%), and Cr (44.2%), highlighting that organic amendments also play a significant role, particularly for Cr and Pb. Factor 3 was overwhelmingly responsible for Hg accumulation, contributing 75.5% of total Hg in surface soils, whereas its contributions to other metals were minor (11–13%). These results quantitatively confirm that Hg is predominantly sourced from atmospheric deposition, likely from industrial emissions and coal combustion, consistent with local emission inventories reporting approximately 292 tons of Hg accumulation in 2014 and 2.4 tons of Hg emission due to coal consumption in Shaanxi Province in 2017.

In subsurface soils (20–40 cm), the relative contributions shifted slightly. Factor 1 contributed 39% of Cu, 50.9% of Zn, 50.9% of As, 51.2% of Pb, and 53.4% of Cr, indicating that inorganic fertilizer inputs also influence deeper layers, possibly through limited leaching or vertical migration. Factor 2 contributed 28.9% of Hg, 45.8% of Cu, 29% of Zn, 31% of As, 28.3% of Pb, and 25.7% of Cr, showing a somewhat higher influence on Cu compared to surface soils. Factor 3 remained the major contributor to Hg in subsurface soils (71.1%) while contributing modestly to other metals (15–21%), reinforcing the dominance of atmospheric deposition for Hg.

Quantitatively, the PMF results indicate that inorganic and organic fertilizers are the primary sources of Cu, Zn, As, Pb, and Cr, together contributing between 70% and 85% of these metals in surface soils. In contrast, atmospheric deposition accounts for over 70% of Hg accumulation at both depths, with fertilizer contributions below 30%. These findings align with previous studies showing strong enrichment of fertilizer-associated metals in top soils, while Hg reflects regional and local atmospheric inputs.

Overall, the PMF-based source apportionment provides a robust quantitative framework for identifying the dominant sources of heavy metals in orchard soils. The results underscore the need for targeted management strategies: minimizing excess application of inorganic and organic fertilizers to control Cu, Zn, Pb, As, and Cr, and monitoring atmospheric Hg deposition to mitigate long-term contamination risks.

## Conclusions

Our findings suggest that long-term cultivation in apple orchards significantly increased the accumulation of heavy metals, particularly Hg, Cu, and Pb. The pollution load of these metals was higher at the surface and cumulative ecological risk of selected heavy metals was at considerable level. Whereas, individual ecological risk of Hg was at moderate level and the other heavy metals were at low ecological risk. Inorganic and organic fertilizers were identified as the primary sources of Cu, Zn, As, Pb, and Cr accumulation, while Hg was associated with atmospheric deposition. The long-term use of inorganic and organic fertilizers, pesticides, and proximity to industrial activities all contributed to the increased heavy metal content in apple orchards. Therefore, it is crucial to implement policies aimed at reducing soil heavy metals level especially Hg pollution, by regulating the use of fertilizers and pesticides inputs for sustainable farming practices. These measures, along with efforts to improve soil quality, will help mitigate heavy metal contamination and reduce ecological risks in apple orchards.

## Supplementary Information

Below is the link to the electronic supplementary material.

Supplementary Material 1

## Data Availability

The dataset used or analyzed during the current study is available from the corresponding author on reasonable request.

## Declarations

This article does not contain any studies with human or animal subjects performed by any of the authors.
