# Supplementary material for: Long-term apple orchard cultivation drives selective accumulation and moderate ecological risk of heavy metals in loess Plateau, China
Source: Sci Rep. 2026 Jan 19;16:5699. doi: 10.1038/s41598-026-36342-3 (PMC12891573; doi:10.1038/s41598-026-36342-3)
Supplement: Supplementary file 1 — Supplementary Material 1 [file 41598_2026_36342_MOESM1_ESM.docx]

**Supplementary data**

**Long-term apple orchard cultivation drives selective accumulation and moderate ecological risk of heavy metals in Loess Plateau, China**

**Haifeng Pan^1^, Zhikun Chen^2^, Guanghua Jing^2^, Weixi Wang^2^, Muhammad Imran^2^, Wenna Bao^3^**

*^1^School of Life and Health Sciences, Huzhou College, Huzhou 313000, China*

*^2^Key Laboratory of Soil Resource & Biotech Application, Xi'an Botanical Garden of Shaanxi Province (Institute of Botany Shaanxi Province), Xi'an 710061, China*

*^3^School of Biological and Chemical Engineering, Zhejiang University of Science and Technology, Hangzhou 310023, China*

**Authors Affiliation and address**

Haifeng Pan: [haifengpan518@163.com](mailto:haifengpan518@163.com), School of Life and Health Sciences, Huzhou College, Huzhou 313000, China

Zhikun Chen: E-mail: zhikunchen@xab.ac.cn, Xi'an Botanical Garden of Shaanxi Province (Institute of Botany Shaanxi Province), Xi’an 710061, China

Guanghua Jing: E-mail: [jghghl@xab.ac.cn](mailto:jghghl@xab.ac.cn), Xi'an Botanical Garden of Shaanxi Province (Institute of Botany of Shaanxi Province), Xi’an 710061, China

Wang Weixi: E-mail: [18829349150@163.com,](mailto:18829349150@163.com,)Xi'an Botanical Garden of Shaanxi Province (Institute of Botany Shaanxi Province), Xi’an 710061, China

Muhammad Imran: E-mail: [Imranm@gudgk.edu.pk](mailto:Imranm@gudgk.edu.pk), Xi'an Botanical Garden of Shaanxi Province (Institute of Botany Shaanxi Province), Xi’an 710061, China

Wenna Bao: E-mail: [wennabao@163.com](mailto:wennabao@163.com) , School of Biological and Chemical Engineering, Zhejiang University of Science and Technology, Hangzhou 310023, China

**Corresponding author**:

Zhikun Chen, E-mail: [zhikunchen@xab.ac.cn](mailto:zhikunchen@xab.ac.cn),Tel: +8618792936387, Fax: 008602961109925

Wenna Bao, E-mail: [wennabao@163.com](mailto:Wennabao@163.com),Tel: +86-571-85070340, Fax: +86-571-85071165


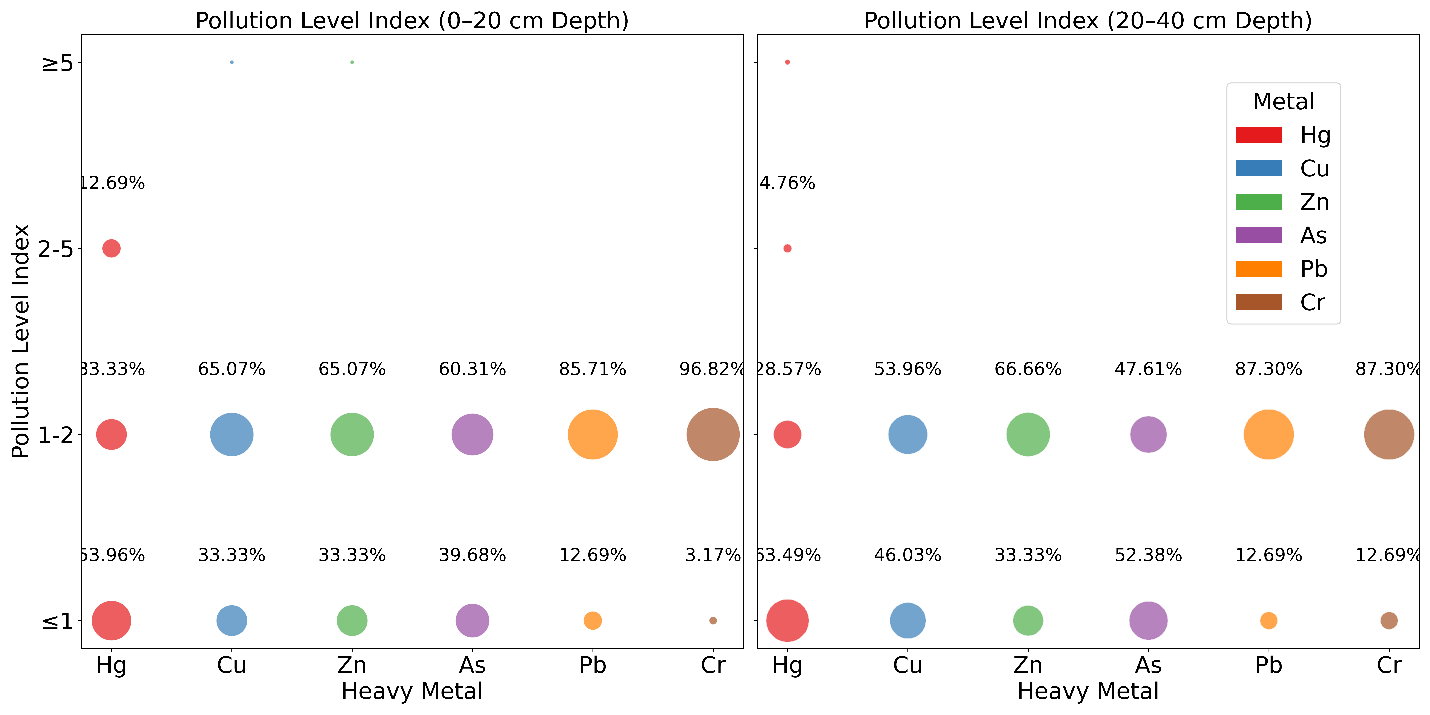


Figure S1. The heavy metals pollution load at different levels


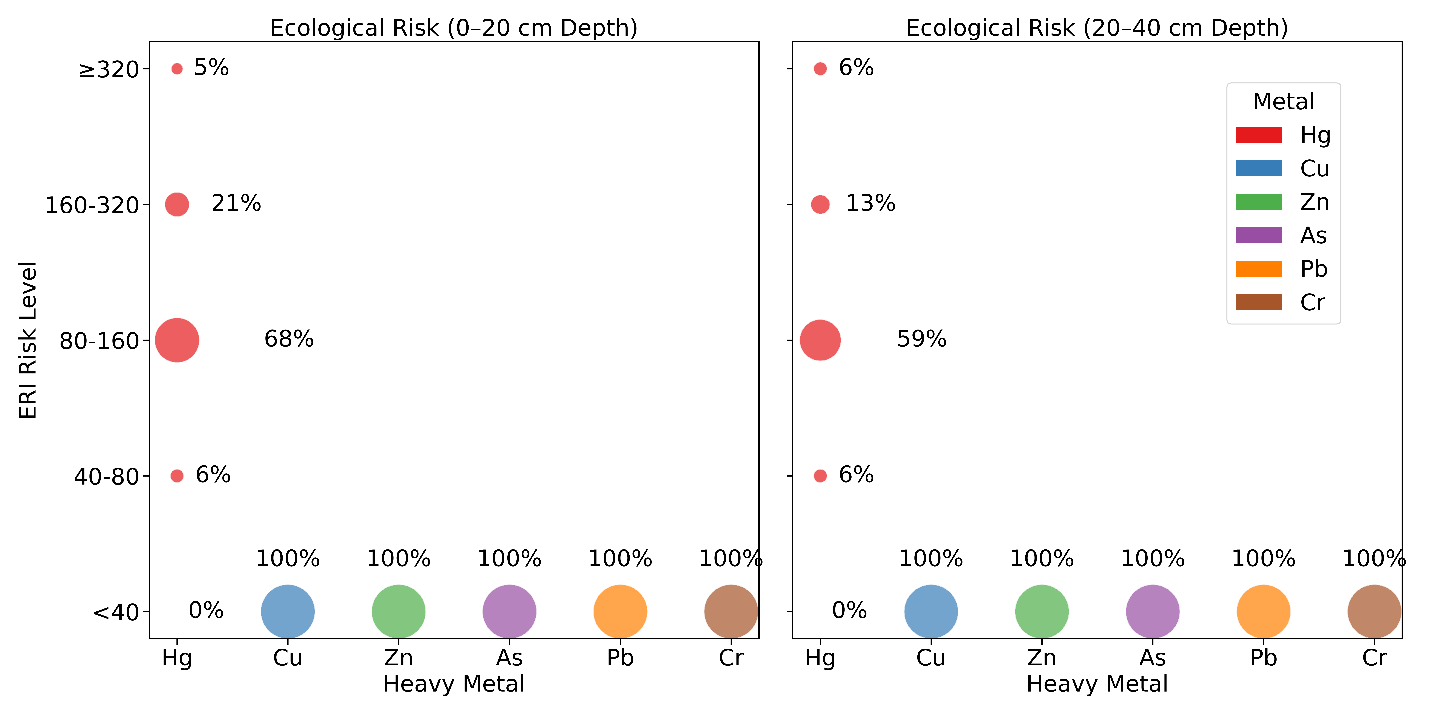


Figure S2. The heavy metals ecological risk at different levels
